# 

**Published:** 1922-03

**Authors:** 


### RECENT APPOINTMENTS.

### Public Health.

Mr. R. J. Allison, M.R.C.S., L.R.C.P., to be Senior Assistant Resident Medical Officer at the County Tuberculosis Sanatorium, Harefield, Middlesex.

Mr. Hugh George Trayer, to be Medical Superintendent at Baguley Sanatorium, Manchester.

Dr. W. S. O'Loughlin, to be Resident Medical Officer for the Institution and Scattered Homes, Romford, Essex.

Dr. F. L. Keith, M.D., Ch.B., D.P.H., to be Assistant School Medical Officer to the County Borough of East Ham Education Committee.

Dr. W. H. Brodie, M.B., B.S., to be V.D. Medical Officer for the County Borough of Darlington.

Miss Evelyn D. Brown, M.B., Ch.B., to be Assistant Medical Officer of Health and School Medical Officer for the County Borough of Bolton.

Dr. S. Eleanor Hill, M.B., B.S., M.D., to be Assistant Medical Officer (Maternity Home and Welfare Clinics) for the Metropolitan Borough of Deptford.

Mr. V. J. F. Lack to be Medical Officer for Maternity and Child Welfare Work for Greenwich.

Mr. D. Dempster, M.B., Ch.B., D.P.H., to be Tuberculosis Officer for the County of Northampton.

Miss Kathleen L. Cass to be Assistant Medical Officer for Lindsey.

Mr. George A. Hayman, M.R.C.S., L.R.C.P.(Lond.), to be Assistant County Tuberculosis Officer for Bedfordshire.

Dr. T. N. V. Potts, M.B., B.S., B.Hy., D.P.H., to be Assistant Tuberculosis Medical Officer for City and County, Newcastle-on-Tyne, and Medical Officer in Charge, City Sanatorium, Newcastle-on-Tyne.

### Institutional.

Mr. H. Ernest Griffiths, M.S., F.R.C.S., and Mr. T. Pomfret Kilner, F.R.C.S., have been appointed Surgeons to the Albert Dock Hospital of the Seamen's Hospital Society.

Mr. J. A. Cowper, late Assistant Secretary of the Royal Northern Hospital (formerly the Great Northern Central Hospital), to be Secretary of the Royal Hospital for Children, Liverpool.

Dr. Ewen J. Maclean, M.D., C.M. (Edin.), to be Professor Extraordinary of Gynæcology and Obstetrics at the Welsh National School of Medicine, Cardiff.

### BOOKS RECEIVED.

"Journal of Comparative Legislation and International Law." October, 1921.

"The Rights of the Ex-Service Man and Woman." By Wilkinson Sherren. (L. J. Gooding, 25, Old Queen Street, Westminster, S.W. 1.; 6d. net.)

"The City Diary, 1922." With a directory of the City of London Corporation and its staff, the municipal and quasi-municipal organisations of the City, &c. (The City Press; 2s. 9d. post free.)

"Guy's Hospital Reports." Vol. 71. (Vol. 1, Fourth Series). No. 4, October, 1921. (Henry Frowde and Hodder & Stoughton; single number 12s. 6d. net, subscription for volume of four numbers, 2 guineas, post free).

"British Medical Association Annual Handbook, 1921-2." (5s. net.)

"Willing's Press Guide, 1922." (James Willing, Ltd.; 2s. 6d. net.) The forty-ninth annual issue of this most useful handbook of the Press of the United Kingdom.

"The Nursing Mirror Pocket Encyclopædia, and Diary 1922." Fifteenth year, enlarged and revised. (The Scientific Press, Ltd.; 1s. 6d. net, 1s. 8d. post free.) The new edition of this well-known pocket reference guide for nurses and midwives.

"Health in Few Words." By R. H. Firth, D.P.H., F.R.C.S. 20th Thousand. (John Bale; 2d.)

### PARLIAMENTARY QUESTIONS AND ANSWERS.

### Blind Welfare (Feb. 13).

Mr. Frederick Roberts asked the Minister of Health what local authorities, if any, had not yet submitted schemes for promoting the welfare of the blind as required by The Blind Persons Act, 1920; to what extent the powers conferred by Clause 2 of the Act had been exercised by local authorities; and what steps his department had taken to urge upon them the necessity for using the powers and fulfilling the duties laid down in the Act.

Sir Alfred Mond said that 51 local authorities have not yet submitted schemes, but 40 of these authorities have schemes in preparation. All the schemes submitted provide for the exercise of the powers contained in Section 2 of the Act. While it has been difficult to exercise pressure in the matter in view of the present financial conditions, every assistance has been offered to local authorities.

### Maternity and Child Welfare Grants (Feb. 9).

Mr. Rhys Davies asked whether the Minister of Health had received protests from public and other bodies against the action of his department relative to the curtailment of assistance to local authorities in the treatment of expectant mothers and children, and whether he was prepared, in view of those protests, to adopt a more generous attitude on the subject.

Sir Alfred Mond stated that protests had been received against a reduction of grant on such services. He hoped that it would be found possible to continue the Exchequer assistance to local authorities so as to enable them to carry on their maternity and child welfare services unimpaired.

### Milk Distribution (Feb. 13).

Mr. Myers asked the Minister of Health whether he could give particulars of the local authorities which distribute milk free for mothers and children, and of those which distribute milk at reduced prices; and whether he could state the conditions laid down by the local authorities under Circular 185 of his department and the prices, if any, charged by them for milk distributed under their auspices.

Sir Alfred Mond replied that of the 416 local authorities carrying out maternity and child welfare schemes, about 400 distribute milk at less than cost price to mothers and children on the conditions laid down by Circular 185. Practically all of them had adopted a scale of income below which milk is supplied free of cost, and about 100 had adopted a further scale within which milk is supplied at half price.

### Asylum Administration (Feb. 9).

Mr. Trevelyan Thomson asked the Minister of Health whether, in view of the difficulties which have arisen in securing some of the evidence necessary to enable his departmental committee to make a complete investigation into the charges made about the treatment of asylum patients, he would reconsider the desirability of recommending the appointment of a Royal Commission with full powers both of investigation into the present system and of recommendation as to the future treatment of all types of mental disorder.

Sir Alfred Mond said that the question was under his consideration, but he pointed out that it would necessarily involve a long delay before any practicable steps could be taken and postpone reforms which by general agreement might, he hoped, be introduced at an early date. In view of the necessity for an expeditious investigation into the allegations made by Dr. Lomax he had appointed a Departmental Committee. As regards the personnel of the committee he was informed that neither of the medical members was or had been associated with any asylum maintained out of public funds, nor was it correct to say that the chairman was in any sense a representative of the system criticised. He feared it was impossible for him to obtain the services of any expert who had not already shown interest in Dr. Lomax's criticisms. He regretted that Dr. Lomax was not prepared to substantiate before the committee the charges which he had publicly made, and that the National Asylum Workers' Union had refused to defend their members against Dr. Lomax's charges; but he could not admit that either he or they were entitled to dictate the composition of any tribunal of inquiry on the issues raised for the appointment of which the Minister of Health was solely responsible.

### KING EDWARD'S HOSPITAL FUND.

### Taxation of Legacies to Hospitals.

At a special meeting of the President and General Council of King Edward's Hospital Fund for London, held at St. James's Palace on February 1, when Lord Donoughmore, member of the President's Powers Committee, was in the chair, reports of the Revenue Committee and the Management Committee were presented, and it was resolved (1) that the King's Fund take steps to put into operation the scheme for a combined public appeal; (2) that it is not desirable to proceed to the institution of a "contributory scheme" until the results which follow the "combined public appeal" are known. In the meantime, steps should be taken to obtain the considered opinion of the medical profession in London, upon whom the duty of carrying any such scheme into practice would ultimately devolve.

The Chairman of the Revenue Committee (Sir Alan Anderson) presented a further report recommending particularly, among the suggestions discussed by Lord Cave's Committee: (*a*) That all subscriptions to hospitals from employers should be allowed as a deduction from profits for the purpose of income-tax; (*b*) that when residuary estate has been bequeathed to a hospital the hospital should be allowed to claim repayment of income-tax as from the expiration of a year from the testator's death; (*c*) that all testamentary gifts to hospitals should be exempted from legacy and succession duties.

The General Council adopted unanimously the recommendation of the Revenue Committee that the Government be urged to take immediate steps to give effect to these recommendations of Lord Cave's Committee.

On the motion of Lord Burnham it was agreed that the Hospital Economy Committee be instructed to consider and report how best to connect and utilise the vacant accommodation and service of Poor Law infirmaries within the area of the fund for the general purposes of hospital work.

### CLEANSING VERMINOUS ARTICLES AND PERSONS.

In its new General Powers Bill, which is to be submitted to Parliament in the session of 1922, the London County Council will seek additional powers for dealing with filthy and verminous articles and premises. The intention is to render more effective the powers already possessed by borough councils under the General Powers Act of 1904. Difficulty has been experienced in administering the 1904 Act, under which a sanitary authority cannot deal with verminous articles or premises unless a borough medical officer can certify that he has actually seen vermin therein. Under the Bill it will be possible for a sanitary authority to deal with such articles upon a report from a medical officer that they had been used by a verminous person. The work of cleansing or destruction will be carried out at the expense of the sanitary authority, and provision is made requiring the sanitary authority to compensate an owner who suffers unnecessary damage by reason of the exercise of the powers of the section, and also to compensate him for articles destroyed. A definition of vermin is included, and improved powers of entry are sought. The Council believes it will be more convenient to repeal all the provisions dealing with the subject in the Act of 1904 than to modify them, and the Bill will therefore contain new provisions.

---

Sir Alfred Mond, Minister of Health, has issued a letter to the Metropolitan Boards of Guardians on the advantage to the sick poor of London from the concentration of rheumatic patients in one institution under the care of a specially qualified consulting physician. St. Marylebone Guardians are prepared to establish an "arthritis unit" for the treatment of such cases, women patients only in the first instance, at their hospital, if they are assured of the support of a sufficient number of other Boards who would send patients. This proposal has the sanction of the Minister of Health. The charge would be £1 11s. 6d. a week.

### OUR PRIZE LEAFLETS.

In dealing with "The Dangers of Alcohol" many of our competitors have not sufficiently realised that their efforts were addressed to elementary and secondary school children. We have received a number of entries which are really total abstinence tracts, filled with highly disputable doctrine. Now the militant teetotaller is as out of place in the schoolroom as the militant politician. As we write, parents are protesting against his appearance as a lecturer in some of the public schools.

The prize of Three Guineas is awarded to *Antialk*, who, despite his uncompromising pseudonym, does not spoil a good case by over-statement. His leaflet, while sufficiently emphatic, is moderate and well expressed. Honourable mention is due to *Odd Number* and to *Chrysanthemum*.

### THE DANGERS OF ALCOHOL.

YOUR BRAIN

controls your body. Without it you can do nothing. It is made up of millions of tiny cells, which communicate with each other by means of very delicate fibres. If a railway line breaks down it causes a great deal of trouble. How much more trouble is caused if the only links between the different parts of your brain are

BLOCKED UP!

Yet this is what is happening every day to the thousands of people who are given to the too liberal use of that most popular and dangerous poison—

ALCOHOL.

Do you want to grow up without *Judgment, Reason or Self-control?* For this is what will become of you if you habitually drink alcohol. Gradually, between the ages of twelve and twenty-five, the most important addition of three new powers is being made to your brain. When you are born your brain is complete except for these three powers.

As you go through life you will meet with temptations; to resist them you must have strong self-control. Also you will have to work: whatever your career is, it will require Judgment and Reason. Nothing is so ruinous to these three powers as alcohol.

Most doctors say that alcohol does no harm to one *taken sparingly*. This is quite true. It is even good for people getting on in years. But almost all agree that it is very harmful taken as a beverage, especially to young people.

A drunkard can move, think and see to a certain extent, but his Judgment, Reason and Self-control are slowly but surely undermined by the alcohol. Think of this when next you are tempted to drink it, and stop yourself in time.

*Antialk.*

### THE GENERAL NURSING COUNCIL FOR ENGLAND AND WALES.

At a meeting of the General Nursing Council on February 3, Sir Wilmot Herringham, K.C.M.G., K.B., presided for the first time since his appointment to succeed Mr. Priestley, all the members who resigned with the late Chairman having withdrawn their resignations. Sir Alfred Mond has stated that these resignations had nothing to do either with the question of the syllabus of training or the question of the entry of certificates in the Register, and that there has been no controversy between the Council and the Minister, relations having been uniformly cordial.

The Chairman read letters from the British Hospitals Association stating that the Syllabus was considered much too elaborate and suggesting that the date of its coming into operation should be deferred for at least one year. The Association regretted that the hospitals as a whole had not had an opportunity of expressing an opinion upon it and hoped that they would be given such an opportunity. The Tynemouth Union, while approving of the ideal created, thought the standard which it sets up impracticable at the present time as demanding too high an educational standard and tending to debar otherwise eligible candidates from the nursing profession. On the other hand, the West Ham Union wrote strongly objecting to any curtailment. The Chartered Society of Massage and Medical Gymnastics asked for a definite statement that the Massage Course is intended to instruct in the principles underlying treatment, and not to qualify for practical work.

Sir Jenner Verrall, in moving a vote of thanks to the former Chairman, which was carried unanimously, remarked that all public bodies, whatever their differences on matters of policy and procedure, were ready to join in expressing their satisfaction in good work done by any member. Mr. Priestley's legal knowledge and ability had been constantly at their service. They would always remember his courtesy, his justice and his devotion.

The Council approved additional sub-sections to Rules 9 (1) and 11 (1), in the case of an application for admission to the general part of the Register, providing that an "Existing Nurse" shall produce a certificate, or certificates, that she has had a conjoint three years' training in hospitals for men only and for women only, approved by the Council, of not less than two years and one year in such hospitals respectively before Nov. 1, 1919, and that an "Intermediate Nurse" shall produce similar certificates before July, 1924.

It was decided that the word "Certificate" should appear in the Register against the name of each nurse possessing that qualification, together with the dates of training and names of hospitals where training was received. Where the name of a hospital has been changed it is to appear in the Register in the form given on the nurse's certificate.

At a further meeting on February 17 it was decided to accept, in place of an original certificate, a duly certified copy thereof by a Justice of the Peace, a barrister or a solicitor; or, where the applicant is a member of any organised body of nurses recognised by the Council, a declaration signed by a responsible officer of that body that on her admission to membership the certificate or a copy thereof was produced and verified.

A "Draft Prescribed Scheme" was approved for the election on the Council of persons registered as nurses. These are to be: Six past or present matrons of training schools for nurses, namely, four matrons of general hospitals (at least one of a provincial general hospital), and two matrons of poor law infirmaries (one a provincial infirmary); and five registered nurses, one of whom must be engaged in the Public Health service; all to be elected by the nurses on the General part of the Register. Also one male nurse, two mental nurses (male and female), one fever nurse and one sick children's nurse; each to be elected by the nurses on the Supplementary parts of the Register of the class to which he or she belongs.

On the motion of Miss Coulton, who represented that some members had not yet had an opportunity of serving on any sub-committee, it was decided that the members of the various sub-committees should go out of office and that an election should take place on February 24. The results had not been issued when we went to press.

The Hospitals Commission desires to make it known that any applications for emergency grants required before the end of the present financial year (March 31) should be made as early as possible. All applications must be made through the local committee for the area.

### MEMORANDA FOR MARCH.

The British Hospitals Association will hold its annual conference on May 25 and 26, at Liverpool. All hospital representatives wishing to attend should notify the hon. secretary as soon as possible.

Medical practitioners are cordially invited to attend a series of clinical lectures now being given at the Cancer Hospital, Fulham Road, S.W., on the diagnosis and treatment of cancer. The series, which began on February 21, will be continued until March 28, on Tuesdays and Fridays, at 4 p.m.

At the request of many firms, the Medical Committee of the Industrial Welfare Society is about to prepare suitable forms for the use of medical officers when making their examination of employees. Dr. Coles, the chairman, invites firms which have had experience in medical work to send him any helpful forms or suggestions. A copy of notes based upon his own experience in industrial medical work will be sent to any who are interested, and he will value comments and criticisms.

The Medical Research Council have appointed the following Committee to advise them upon the promotion of researches into the biological action of light, with a view to obtaining better knowledge of the actions of sunlight and other forms of light upon the human body in health or disease: Professor W. M. Bayliss, F.R.S. (chairman), Mr. J. E. Barnard, Dr. H. H. Dale, F.R.S., Captain S. R. Douglas (late I.M.S.), Sir Henry Gauvain, M.D., Dr. Leonard Hill, F.R.S., and Dr. J. H. Sequeira, F.R.C.P., Dr. Edgar Schuster (secretary).

ROYAL SANITARY INSTITUTE.—Our readers are reminded that Part II. of the Spring Term course of lectures for sanitary officers will begin on March 30. Inspections and demonstrations are arranged, and include visits to public and private works illustrative of sanitary practice and administration. Part II. of the course for women health visitors and child-welfare workers will begin on March 27. The fee for Part II. is 2 guineas in each case. For particulars, apply to the Secretary, Royal Sanitary Institute, 90, Buckingham Palace Road, S.W. 1.

We are informed by the Registry of Friendly Societies that a revised list of societies registered under the Friendly Societies Act, 1896, can now be purchased through any bookseller or direct from H.M. Stationery Office. The list contains, in addition to registered names and offices, particulars of the membership and funds of each society as set out in its annual return for 1919. It is issued in eight sections, Nos. I.—VI. relating to England, No. VII. to Wales, and No. VIII. to Scotland. In ordering the list the district for which it is required should be stated.

A series of eight lectures, arranged by the People's League of Health, is in course of delivery at the Royal Society of Arts, John Street, Adelphi, W.C.2, on Mondays at 6 p.m., on "The Mind and What We Ought to Know about It." The subjects during March are as follows: "Fatigue and Sleep" (Sir R. Armstrong Jones, M.D.), March 6; "Mind and Body" (Sir F. Mott, M.D.), March 13; "Crime and Delinquency" (Dr. W. A. Potts), March 20; "Mental Deficiency" (Dr. Tredgold), March 27. Tickets can be obtained from the Hon. Organisers, People's League of Health, 12 Stratford Place, W.1.

At the request of the Ramsay Memorial Committee a commemorative medal of the late Professor Sir William Ramsay, K.C.B., F.R.S., has been executed by the distinguished French sculptor, M. Louis Bottée and will be struck in London probably during April, when it is known approximately how many will be required. The price of the bronze medal, including postage, will be (*a*) to subscribers to the Ramsay Fund, 5s.; (*b*) to all other persons, 7s. 6d. The appropriate remittance should be sent by cheque or postal order to Dr. Walter W. Seton, Organising Secretary, Ramsay Memorial Fund, University College, London, Gower Street W.C. 1, before February 28, after which date the price will be increased. Acknowledgment of the order will be sent only if a stamped addressed envelope is enclosed for the purpose.

